# A radiological and pathological analysis of screen-detected and interval-detected breast cancers in Belfast

**DOI:** 10.1186/bcr3307

**Published:** 2012-11-09

**Authors:** J Malloy, C Hennell, L Bamford, K Lowry, L Tong

**Affiliations:** 1 Belfast City Hospital, Belfast, UK; 2 Northern Ireland Breast Screening Programme (Eastern Unit), Belfast, UK

## Introduction

The Belfast Breast Screening Programme serves a population of approximately 25,000 patients. We aimed to analyse radiological and pathological trends between screen-detected and interval breast cancers, and determine our screening lesion miss rate.

## Methods

Using the Quality Assurance Reference Centre (QARC) database patients were identified with screen-detected or interval breast cancers diagnosed via the Belfast Breast Screening Programme over a fixed period. Film packs and operative specimen reports were analysed for radiological and pathological data.

## Results

Screening identified 75% of breast cancers. Only 30% of screen-detected lesions were palpable by breast clinicians. Sixty-one per cent of lesions were in the upper outer quadrant, with equal left-right distribution. Radiological measurements underestimated lesion size by 22%. There was moderate correlation between lesion size and lymph node status. No other correlations were identified. Twenty-three per cent of interval cancers presented in year 1 following screening, 28% in year 2 and 49% in year 3. They were larger at presentation than screen-detected cancers (29.4 mm vs. 18.2 mm mean size) and of pathologically higher grade (39% vs. 13% grade III). Screen-detected cancers were mostly IDC (63%) or DCIS (18%) subtype. Interval lesions were predominantly IDC subtype (87%). Interval lesions showed more nodes positive per patient. Retrospective review of past screening films of interval-detected cancers showed suspicious features present in 17% of cases.

## Conclusion

Breast screening identifies three-quarters of breast cancers in the screening population. Interval cancers present with increasing frequency through the screening cycle and are faster growing, pathologically more aggressive lesions.

